# Dichotomous Effects of Mu Opioid Receptor Activation on Striatal Low-Threshold Spike Interneurons

**DOI:** 10.3389/fncel.2017.00385

**Published:** 2017-12-05

**Authors:** Rasha Elghaba, Enrico Bracci

**Affiliations:** Department of Psychology, The University of Sheffield, Sheffield, United Kingdom

**Keywords:** striatun, interneuron, nitric oxide, opioids, acetylcholine, inhibition (psychology), excitation, GABA

## Abstract

Striatal low-threshold spike interneurons (LTSIs) are tonically active neurons that express GABA and nitric oxide synthase and are involved in information processing as well as neurovascular coupling. While mu opioid receptors (MORs) and their ligand encephalin are prominent in the striatum, their action on LTSIs has not been investigated. We addressed this issue carrying out whole-cell recordings in transgenic mice in which the NPY-expressing neurons are marked with green fluorescent protein (GFP). The MOR agonist (D-Ala(2), N-MePhe(4), Gly-ol)-enkephalin (DAMGO) produced dual effects on subpopulations of LTSIs. DAMGO caused inhibitory effects, accompanied by decreases of spontaneous firing, in 62% of LTSIs, while depolarizing effects (accompanied by an increase in spontaneous firing) were observed in 23% of LTSIs tested. The dual effects of DAMGO persisted in the presence of tetrodotoxin (TTX), a sodium channel blocker or in the presence of the nicotinic acetylcholine receptor antagonist mecamylamine. However, in the presence of either the GABA_A_ receptor antagonist picrotoxin or the muscarinic cholinergic receptor antagonist atropine, DAMGO only elicited inhibitory effects on LTSIs. Furthermore, we found that DAMGO decreased the amplitude and frequency of spontaneous GABAergic events. Unexpectedly, these effects of DAMGO on spontaneous GABAergic events disappeared after blocking of the muscarinic and nicotinic cholinergic blockers, showing that GABA inputs to LTSIs are not directly modulated by presynaptic MORs. These finding suggest that activation of MORs affect LTSIs both directly and indirectly, through modulation of GABAergic and cholinergic tones. The complex balance between direct and indirect effects determines the net effect of DAMGO on LTSIs.

## Introduction

Striatal low-threshold spike interneurons (LTSIs) are GABAergic cells that were originally identified by Kawaguchi and colleagues as cells expressing neuropeptide Y (NPY), somatostatin and nitric oxide synthase (Kawaguchi, 1993; Kubota and Kawaguchi, 2000). They were shown to have long and relatively sparse dendritic and axonal arborizations and to be capable of generating calcium channel-mediated plateau potentials in response to depolarizations or, as a rebound, after the end of a hyperpolarizing step (Kawaguchi, 1993). The relative rarity of these cells has initially impaired systematic explorations of their properties and function. However, nitric oxide-triggered intracellular cascades were found to be necessary for the expression of long term plasticity at corticostriatal synapses (Centonze et al., 1999). The introduction of transgenic mice expressing green fluorescent protein (GFP) in NPY-expressing neurons or Cre recombinase in somatostatin- or NOS-expressing neurons, has allowed more extensive investigations of LTSIs (Ibáñez-Sandoval et al., 2011; Rafalovich et al., 2015). Using such mice, it was recently shown that LTSIs play a specialized GABAergic inhibitory role in the striatum, that is starkly different from the one played by parvalbumin-expressing fast spiking interneurons. While fast spiking interneurons target proximal dendrites and somata of nearby projection neurons (and do not synapse on cholinergic interneurons), LTSIs selectively target the distal dendrites of projection neurons located farther away from their own cell bodies (Straub et al., 2016). The LTSIs also form functional GABAergic synapses on cholinergic interneurons (Elghaba et al., 2016; Straub et al., 2016). Optogenetic stimulation of LTSIs was shown to induce long-term depression of corticostriatal synapses impinging on striatal projection neurons, confirming a crucial role for these interneurons in controlling synaptic plasticity (Rafalovich et al., 2015). LTSIs are differentially modulated by dopamine and serotonin (Centonze et al., 2002; Cains et al., 2012). However, little is known about the ability of opioid receptors to control the excitability of these interneurons. In particular, mu opioid receptors (MORs) are prominent in the striatum, being abundantly expressed and mediating the effects of the neuropeptide enkephalin, that is released by projection neurons expressing D1 dopamine receptors (Miura et al., 2007, 2008; Blomeley and Bracci, 2011; Atwood et al., 2014). This issue is of interest, because, if MOR controlled the activity of LTSIs, a reciprocal control between these cells and D1-expressing projection neurons could be present. Furthermore, both nitric oxide (Rafalovich et al., 2015) and enkephalin (Atwood et al., 2014) have been shown to induce long term depression in the striatum and it is therefore important to unravel the interactions between nitrergic and opioidergic neurons. Here, we used transgenic mice expressing GFP in LTSIs to investigate the effects of MOR activation on the activity of LTSIs.

## Materials and Methods

### Animals

Brain slices were obtained from P16–P32 heterozygous BAC NPY-GFP mice in which a humanized Renilla GFP (hrGFP, Stratagene) sequence was inserted into the start of the transitional site of the neuropeptide Y (NPY) gene (Stock, 006417, Jackson Laboratory, Bar Harbor, ME, USA). This colony was bred by crossing the hemizygous BAC-NPY transgenic male mice with wild-type (CD57) females. All animals were bred and housed in the Biological Services Facility, University of Sheffield.

### Slice Preparation

Mice of both sexes were killed and all experiments were carried out by cervical dislocation in accordance with the 1986 Animal (Scientific Procedures) Act, and with approval from the UK Home Office and the Ethical Committee of the University of Sheffield. Following decapitation, the brain was quickly removed from the skull and transferred to a vibrating microtome 7000 cutting chamber (Camden instruments) filled with oxygenated sucrose solution containing (in mM): 184 Sucrose, 2.5 KCl, 1.2 NaH_2_PO_4_, 30 NaHCO_3_, 20 HEPES, 25 Glucose, 5 Sodium ascorbate, 2 Thiourea, 3 sodium pyruvate, 10 MgSO_4_.7H_2_O, 0.5 CaCl_2_.2H_2_O at pH7.4 at 2–5°C continuously bubbled with a carbogen mixture of 95% O_2_ and 5% CO_2_ gas. Sagittal brain slices (250 μm thick) were prepared and immediately transferred to a recovery chamber filled with oxygenated Tris-HEPES recovery solution containing (in mM): 76 TrisHCl, 19.5 Tris-Base, 2.5 KCl, 1.2 NaH_2_PO_4_, 30 NaHCO_3_, 20 HEPES, 25 Glucose, 5 Sodium ascorbate, 2 Thiourea, 3 sodium pyruvate, 10 MgSO_4_.7H_2_O, 0.5 CaCl_2_.2H_2_O at pH 7.3–7.4. Slices were kept at 26°C for 30 min. Then, slices were transferred to a storage chamber filled with oxygenated standard aCSF containing (in mM): 124 NaCl, 3 KCl, 1.2 NaH_2_PO_4_, 26 NaHCO_3_, 15 Glucose, 2 MgSO_4_.7H_2_O, 2 CaCl_2_.2H_2_O at pH 7.3–7.4 for another 30 min. For recordings, slices were placed in a recording chamber where the slices were continuously superfused with oxygenated standard aCSF (flow rate 1.5–2 ml/min) at room temperature (21–24°C). Slices were visualized using an infrared/differential interface contrast microscopy with a 40× water-immersion objective. For visualization of GFP-expressing neurons, we used epifluorescence with standard GFP filters; GFP excitation was provided through a high power blue light LED driver (DC2100, ThorLabs) using constant current mode which provides a constant non-modulated LED current.

### Electrophysiological Recording

For whole-cell recordings, patch pipettes (4–6 MΩ) were prepared by pulling borosilicate glass tubes with a PC-10 puller (Narishige). Pipettes were filled with an intracellular solution containing in (mM): 120 K-Gluconate, 20 KCl, 2 MgCl_2_, 12 HEPES, 0.4 Na-GTP and 4 Na_2_-ATP, 0.04 EGTA, adjusted to pH 7.3 with KOH. Current-clamp recordings were performed in bridge mode using an NPI BA-1S bridge amplifier. For experiments in which spontaneous GABAergic IPSPs were studied, a high-chloride intracellular solution (in which equimolar KCl replaced K-Gluconate) was used to increase chloride driving force and therefore GABAergic responses (Elghaba et al., 2016). Cell-attached recordings were obtained with similar procedure to whole-cell recordings but the membrane was not ruptured. Cholinergic interneurons were identified based on their large somata, lack of fluorescence and presence of spontaneous firing that was detected as rapid biphasic deflections of the recorded potential (Bennett and Wilson, 1999). For perforated whole-cell recordings, 3 mg of amphotericin B were dissolved in 50 μl DMSO to get 60 mg/ml stock solution. Then 20 μl of the stock solution were added to 5 ml of intracellular solution to reach a final concentration of 240 μg/ml. The tip of the patching pipette was usually filled with amphotericin B-free intracellular solution to help the seal formation. Once a seal >1 GΩ was obtained, the cell was left for 10–15 min to allow the amphotericin B pores to be formed. The perforation was usually complete within 15 min from the seal formation. The full perforation was confirmed by observation of action potentials >50 mV and of electrode resistance <50 MΩ.

In NPY-GFP mice, NPY-expressing neurons also express GFP. In the striatum, these GFP-positive cells belong to two distinct interneuronal types: low threshold spike interneurons (LTSIs) and neurogliaform interneurons (NGFIs; Ibáñez-Sandoval et al., 2011; Logie et al., 2013; Elghaba et al., 2016). NPY-positive LTSIs were initially identified based on their fluorescence (usually slightly fainter than that of NGFIs; Ibáñez-Sandoval et al., 2011); once a whole-cell configuration was established, identification of LTSIs was confirmed based on their distinctive electrophysiological properties, that include spontaneous firing and rebound calcium spikes (Kawaguchi, 1993; Beatty et al., 2012; Cains et al., 2012). The neurons’ electrophysiological properties were tested in absence of any drug. We only used neurons with typical and stable LTS properties (Kawaguchi, 1993; Beatty et al., 2012; Cains et al., 2012). When input resistance changed by more than 25% during an experiment (not due to drug application), the cell was discarded.

Spontaneous GABAergic IPSPs were recorded in the presence of the NMDA receptor antagonist D-AP5 (10 μM) and the AMPA receptor antagonist NBQX (10 μM), in order to block ionotropic glutamate transmission. During the recordings of the spontaneous IPSPs, the cell membrane potential was kept at around −80 mV through the whole experiment by manually injecting negative current into the cell. Whenever, an applied drug depolarized or hyperpolarized the cell, the amount of the injected negative current was adjusted to keep the membrane potential at −80 mV. Spontaneous GABAergic IPSPs were defined as fast upward deflections exceeding a threshold of twice the standard deviations (SD) of the baseline noise.

### Drugs

All drugs were obtained from Tocris Biosciences (UK) except Amphotericin B that was obtained from Sigma. Drugs were prepared from dissolved stock solutions. Drugs were applied by adding the appropriate amount to the superfusing solution and reached the slice within >2 min from start of application through a gravity system. Each pharmacological treatment was applied for at least 10 min. The effects of drug(s) were assessed starting 3 min after the start of their application.

### Data Analysis

Data were acquired using Signal (5) software and a Micro 1401 data acquisition unit (C.E.D., UK). Off-line data analysis was carried out with Signal 5 and Spike 2 software.

Changes in spontaneous firing or spontaneous synaptic events induced by pharmacological treatments were assessed by comparing the inter-spike interval (ISI), inter-event interval and event amplitude over periods of >5 min before and during the application measurements started 3 min after the start of the drug application, to allow equilibration of the ligands.

In the experiments monitoring spontaneous GABAergic activity, cumulative frequency and amplitude were calculated in control and during drug applications.

Unless otherwise specified, Student’s unpaired *t*-test was used for statistical comparisons. Statistical significance was accepted if *p* < 0.05.

## Results

### Dual Effects of DAMGO on LTSIs

A total of 83 NPY-GFP mice (both sexes) aged 24 ± 8 days were used in this study. We recorded from 90 striatal LTSIs (82 in whole-cell configurations and eight in perforated-patch configuration) and seven striatal cholinergic interneurons (in cell-attached configuration). The results obtained in LTSIs with whole-cell and perforated-patch techniques were similar. Therefore, data obtained with these techniques were pooled together for analysis.

**Figure 1:**
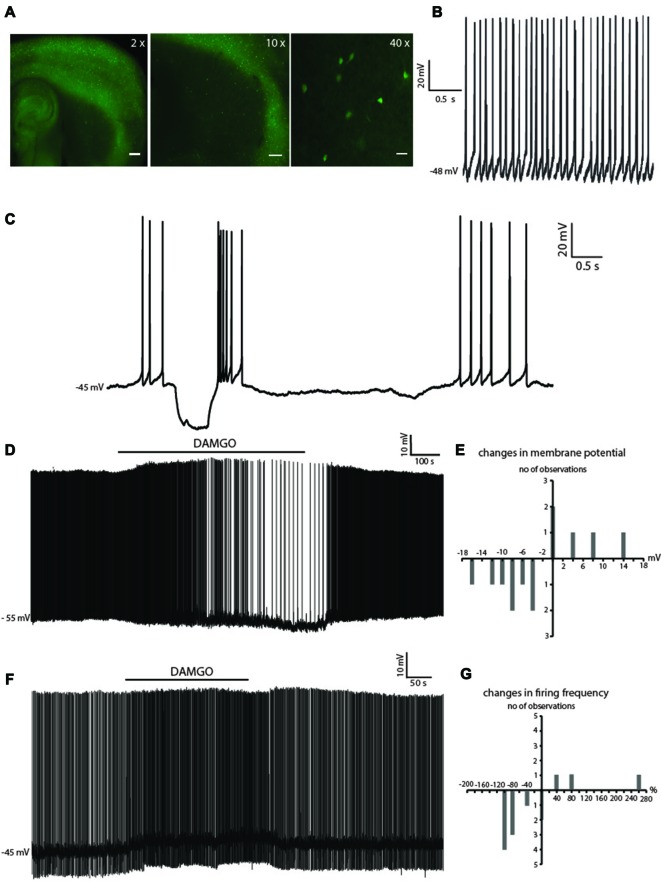
Dichotomous effects of (D-Ala(2), N-MePhe(4), Gly-ol)-enkephalin (DAMGO) on low-threshold spike interneurons (LTSIs). **(A)** Fluorescent photomicrography of a sagittal brain slice from a npy-green fluorescent protein (GFP) mouse. GFP-positive neurons are visible both in the cortex (at higher density) and in the striatum. The right-hand panel shows some GFP-positive neurons in the striatum. These are mainly medium-sized cells, with a number of primary dendrites emerging from the soma. Calibration bars: 500 μm (left panel), 200 μm (middle panel), 40 μm (right panel). **(B)** An example of regular firing observed in a spontaneously active LTS interneuron. **(C)** Negative current injections in a LTSI were followed by prolonged rebound plateau potentials, accompanied by spikes. **(D)** An LTSI, was reversibly hyperpolarized by DAMGO and this was accompanied by decreased firing. **(E)** Distribution of changes in membrane potential observed in 13 LTSIs during DAMGO application. Eight LTSIs were hyperpolarized, three LTSIs were depolarized and two LTSIs were not affected by DAMGO. **(F)** Another LTSI was reversibly depolarized by DAMGO, and its firing was increased during the application. **(G)** Distribution of percentage changes in firing frequency observed in 13 spontaneously active LTSIs during application of DAMGO. DAMGO significantly increased firing frequency in eight LTSIs, significantly decreased firing frequency in three LTSIs DAMGO, did not affect firing in the remaining two LTSIs.

LTSIs were initially identified through their epifluorescence with standard GFP filters (Figure 1A). After patching, LTSIs identification was confirmed based on their distinctive electrophysiological features including spontaneous firing activity, high input resistance (>500 MΩ), relatively depolarized resting membrane potential and ability to generate rebound low-threshold slow spikes in response to negative current injections (Kawaguchi, 1993; Koós and Tepper, 1999; Elghaba et al., 2016). Examples of these properties are shown in Figures 1B,C.

Application of the μ receptor agonist DAMGO (1 μM) caused opposite effects in subpopulations of LTSIs. In 8/13 LTSIs, DAMGO caused hyperpolarizations accompanied by significant (*p* < 0.05) decrease in spontaneous firing frequency (Figure 1D). On the other hand, in 3/13 LTSIs, DAMGO caused depolarizations associated with significant increases in spontaneous firing frequency (*p* < 0.05 for ISI), as shown in Figure 1F). In 2/13 experiments, there were no significant changes in spontaneous firing and the membrane potential was unaffected. The dichotomous effects of DAMGO on membrane potential and spontaneous firing frequency are quantified in Figures 1E,G. No significant correlation was found between firing frequency in control solution and the effects of DAMGO on firing frequency (assessed by Pearson’s correlation coefficient for initial frequency against percentage change).

### DAMGO Inhibits Cholinergic Interneurons

In our previous study we showed that there is a mutual excitatory interaction between LTSIs and cholinergic interneurons in the striatum (Elghaba et al., 2016). Furthermore, a previous study suggested that DAMGO has a powerful inhibitory effect on striatal cholinergic interneurons (Ponterio et al., 2013). Therefore, we investigated if ACh played a role in the dual effect of DAMGO on LTSIs.

**Figure 2:**
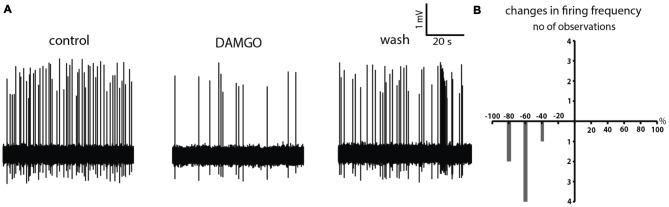
DAMGO inhibits cholinergic interneurons. **(A)** DAMGO application caused a reversible decrease in the spontaneous firing of a cholinergic interneuron recorded in cell-attached configuration. This decrease was reversed by DAMGO washout. **(B)** Distribution of percentage changes in firing frequency observed in seven spontaneously active cholinergic interneurons during application of DAMGO.

We first confirmed the previously published effects of DAMGO on cholinergic interneurons (Ponterio et al., 2013). Given the tendency of cholinergic interneurons’ properties to deteriorate during prolonged whole-cell recordings (Blomeley and Bracci, 2005), we carried out cell-attached recordings from these cells. Cholinergic interneurons were initially identified by their large soma (Wilson et al., 1990), and absence of epifluorescence. Once the cell-attached configuration was established, the identification of cholinergic interneurons was confirmed by the presence of spontaneous firing. In 7/7 cholinergic interneurons, DAMGO caused a significant decrease in spontaneous firing frequency (*p* < 0.05 for ISI; Figure 2A). These results are quantified in Figure 2B.

### Effects of DAMGO on LTSIs in the Presence of TTX

**Figure 3:**
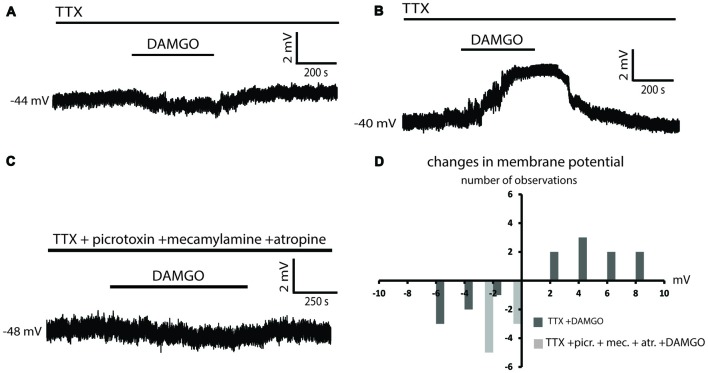
Effects of DAMGO on LTSIs in the presence of tetrodotoxin (TTX). **(A)** An LTS interneuron was reversibily hyperpolarized by DAMGO application in the presence of TTX. **(B)** Another LTS interneuron was reversibly depolarized by DAMGO application in the presence of TTX. **(C)** An LTSI was reversibly hyperpolarized after application of DAMGO in presence of TTX, picrotoxin and cholinergic receptors blockers. **(D)** Distribution of changes in membrane potential observed in 15 LTSIs during application of DAMGO in the presence of TTX alone (nine LTSIs were significantly depolarized, and six LTSIs were significantly hyperpolarized), and in eight LTSIs in the presence of TTX, picrotoxin, and cholinergic blockers (five LTSIs were significantly hyperpolarized, and three LTSIs showed no significant change in their membrane potentials).

In order to cast light on the dual effects of DAMGO on LTSIs, we investigated the effects of DAMGO when applied in the presence of the sodium channel blocker tetrodotoxin (TTX; 10 μM). Under this condition, DAMGO still elicited a dual effect on LTSIs. In 10/15 LTSI, DAMGO elicited depolarizing effects, while in 5/15 LTSI it caused hyperpolarizations (Figure 3). The proportion of depolarization caused by DAMGO in the presence of TTX (66.7%) was significantly larger than that observed in control solution (15.38%; *p* = 0.007; *N-1 Chi-squared* test for binomial distributions).

In a recent study we found that LTSIs are affected by both GABAergic and cholinergic tones (Elghaba et al., 2016). These tonic influences can persist even in the presence of TTX. Therefore, we studied the effects of DAMGO in the presence of GABA and ACh receptor blockers. We applied DAMGO in the presence of TTX (1 μM), the GABA_A_ receptor blocker picrotoxin (100 μM), the nicotinic receptor blocker mecamylamine (10 μM) and the muscarinic receptor blocker atropine (20 μM). Under these conditions, DAMGO did not cause any depolarizing response in LTSIs. Hyperpolarizations were observed in 5/8 LTSIs while in 3/8 LTSIs DAMGO did not affect the membrane potential. The results of these experiments are quantified in Figure 3D. We concluded that GABAergic and/or cholinergic tones are required for the depolarizing effects caused by DAMGO on some LTSIs.

### Effects of DAMGO on LTSIs in the Presence of Picrotoxin and Cholinergic Receptor Antagonists

**Figure 4:**
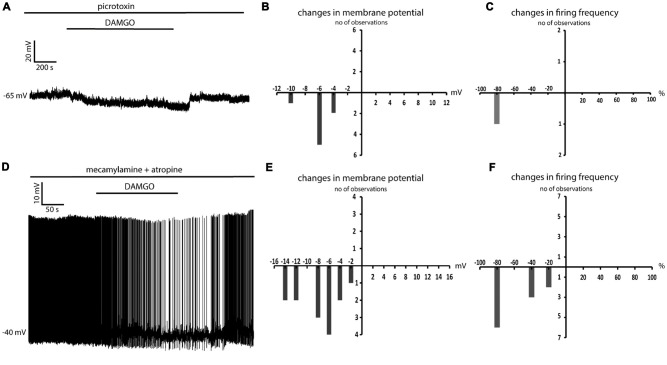
Effects of DAMGO on LTS interneuron in the presence of picrotoxin or cholinergic receptors antagonists. **(A)** In the presence of picrotoxin, DAMGO reversibly hyperpolarized an LTS interneuron. **(B)** Distribution of changes in membrane potential in eight LTSIs during DAMGO application in the presence of picrotoxin. **(C)** In presence of picrotoxin. DAMGO significantly decreased the firing frequency in 1/1 LTS interneuron. **(D)** In the presence of cholinergic receptors antagonists, DAMGO caused a reversible hyperpolarization in a LTSI. **(E)** Distribution of changes in membrane potential in 14 LTSIs after application of DAMGO in the presence of cholinergic receptors antagonists. **(F)** Distribution of changes in the firing frequency in 11 spontaneously active LTSIs after application of DAMGO in the presence of cholinergic receptors antagonists. DAMGO significantly decreased the firing frequency in 11 LTS interneuron.

In order to test whether both GABAergic and cholinergic tonic influences were required for the depolarizing effects of DAMGO, we applied DAMGO in the presence of picrotoxin alone. In 8/8 experiments, DAMGO caused hyperpolarization of LTSIs (Figures 4A,B). Only 1/8 LTSI was spontaneously active. In this interneuron, DAMGO significantly decreased the firing frequency of that LTSIs (Figure 4C). Furthermore, application of DAMGO in the presence of atropine and mecamylamine also caused hyperpolarizations in 14/14 LTSIs (Figures 4D,E) and significant decrease in the firing frequency (*p* < 0.05 for ISI; Figure 4F) of 11/11 spontaneously active LTSIs. These observations suggest that intact cholinergic and GABAergic tones are required for the depolarizing effects of DAMGO on LTSIs.

### Effects of DAMGO on LTSIs in the Presence of Individual Cholinergic Receptor Antagonists

**Figure 5:**
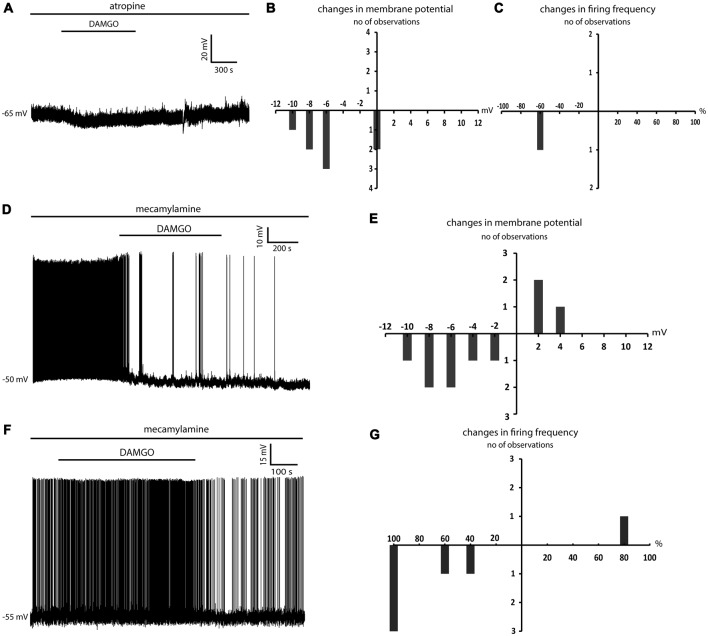
Atropine but not mecamylamine abolished the depolarizing effects of DAMGO. **(A)** Application of DAMGO in the presence of a muscarinic receptor antagonist (atropine) produced a reversible hyperpolarization of an LTSI. This effect was reversed after washout. **(B)** Distribution of changes in the membrane potential induced by DAMGO in eight LTSIs in the presence of atropine. **(C)** Changes in the firing frequency induced by DAMGO in one spontaneously active LTSI in the presence of atropine. **(D)** Application of DAMGO in the presence of mecamylamine produced a reversible hyperpolarization of an LTSI. **(E)** Distribution of changes in the membrane potential induced by DAMGO in 10 LTSIs in the presence of mecamylamine. **(F)** Another LTSI was reversibly depolarized after DAMGO application in the presence of mecamylamine. This depolarization was associated with increased firing. **(G)** Distribution of changes in the firing frequency induced by DAMGO in six spontaneously active LTSIs in the presence of mecamylamine. DAMGO significantly increased firing frequency in one LTSI and significantly decreased firing frequency in five LTSIs.

To further dissect out the individual role of muscarinic and nicotinic receptors, we applied DAMGO in the presence of either atropine or mecamylamine alone. In the presence of atropine, DAMGO caused significant hyperpolarizations in 6/8 LTSIs, one of which was spontaneously active (DAMGO significantly decreased in the firing frequency in this cell; *p* < 0.05 for ISI). No effects were observed in the remaining 2/8 LTSIs. The results are illustrated in Figures 5A–C.

In the presence mecamylamine, DAMGO caused hyperpolarization in 7/10 LTSIs, but 3/10 LTSIs were depolarized by DAMGO (Figures 5D–F). Moreover, DAMGO significantly decrease the firing frequency in 5/6 spontaneously active LTSIs (*p* < 0.05 for ISI), but significantly increased the firing frequency in 1/6 LTSIs (*p* < 0.05 for ISI; Figures 5D,E,G). We concluded that muscarinic component of the cholinergic tone, but not the nicotinic one, is necessary for the depolarizing effects of DAMGO on LTSIs.

### DAMGO Decreases Spontaneous GABAergic Events in LTSIs

Since the effects of DAMGO on tonic GABA levels could not be measured directly, we investigated the effects on DAMGO on the spontaneous GABAergic events detected in LTSIs. In these experiments, a high-chloride internal solution was used to shift GABA_A_ receptor reversal potential towards 0 mV and increase the amplitude of GABAergic events (Gallagher et al., 1978; Houston et al., 2009). LTSI membrane potentials were kept at hyperpolarized levels (between −80 mV and −90 mV) by steady current injections. Changes in membrane potential induced by DAMGO were compensated manually, so that the membrane potential was maintained at control level throughout the experiment.

**Figure 6:**
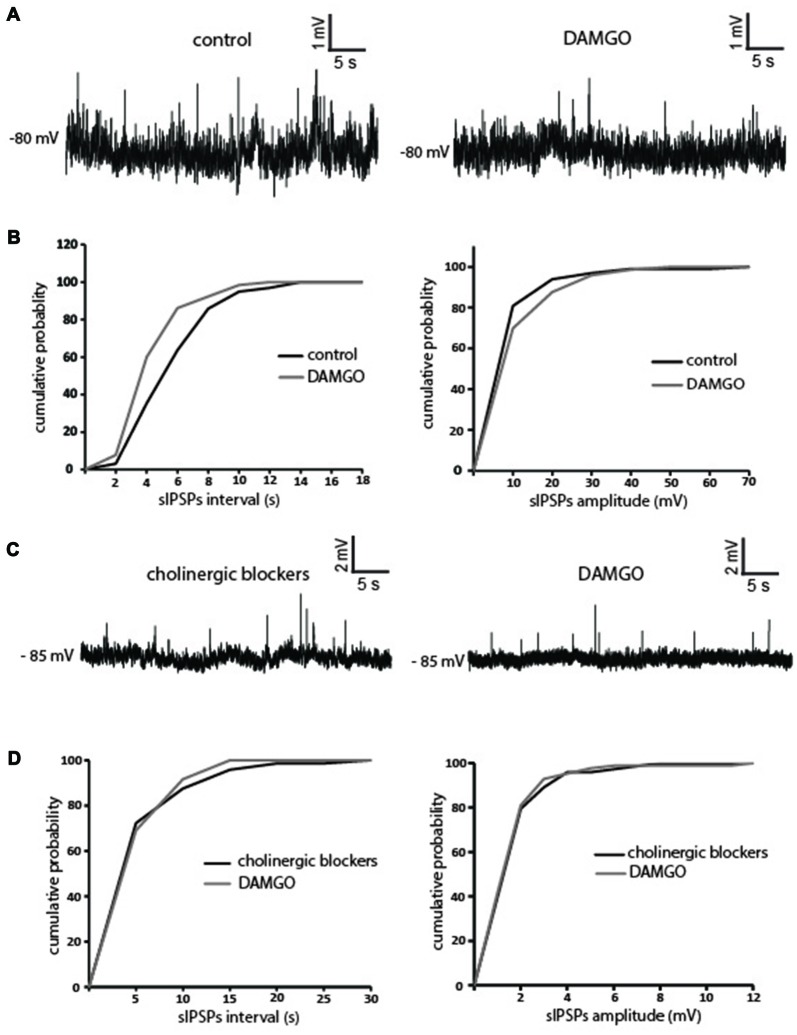
DAMGO decreases GABAergic inputs to LTSIs by reducing the cholinergic tone. **(A)** DAMGO decreased the frequency and the amplitude of spontaneous GABAergic events in an LTSI. **(B)** Quantification of the effects of DAMGO on the frequency and the amplitude of spontaneous GABAergic events in the interneuron of **(A)**. The frequency and the amplitude of spontaneous inhibitory postsynaptic potentials (IPSPs) was significantly (*p* < 0.05 for inter-event intervals and amplitude respectively) decreased by DAMGO. **(C)** In the presence of mecamylamine and atropine, DAMGO did not change the frequency or the amplitude of spontaneous GABAergic events in an LTS interneuron. **(D)** Quantification of the effects of DAMGO on the frequency and the amplitude of spontaneous GABAergic events in the interneuron of **(C)**. Neither the frequency nor the amplitude of spontaneous IPSPs was significantly changed after DAMGO application in the presence of cholinergic blockers.

DAMGO significantly (*p* < 0.05) increased the average inter event interval and decreased the average amplitude of spontaneous GABAergic events in 4/4 LTSIs (Figures 6A,B). In order to determine the possible contribution of cholinergic processes to this modulation, we then applied DAMGO in the presence of mecamylamine and atropine, Surprisingly, in the presence of these cholinergic blockers, DAMGO did not cause any significant change in the frequency and/or of the spontaneous GABAergic events in 4/4 LTSIs (Figures 6C,D). We concluded that there is no direct modulation of DAMGO on GABAergic inputs to LTSIs. Instead, DAMGO reduced GABAergic inputs to LTSIs indirectly through modulation of cholinergic inputs to LTSIs.

## Discussion

**Figure 7:**
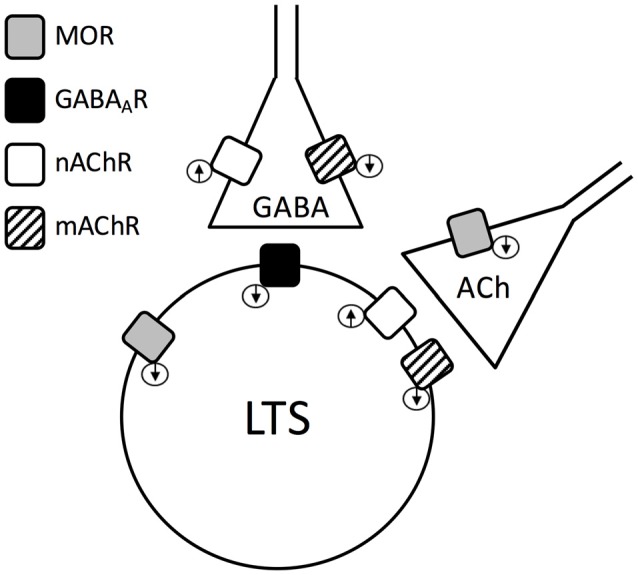
Minimal mechanistic model required to explain the experimental data. The present findings show that an LTSI has GABA_A_, MOR, muscarinic and nicotinic receptors and the state of activation of these postsynaptic receptors affect its activity directly (with GABA_A_, MOR and muscarinic receptors having inhibitory actions, and nicotinic receptors having an excitatory influence). However, GABAergic inputs to an LTSI are also inhibited by muscarinic receptors and facilitated by nicotinic receptors. Furthermore, cholinergic terminals are inhibited by MORs. The complex and variable balance of these interactions has to be considered to explain the dual action of DAMGO on LTSIs (see “Discussion” section for details).

The main conclusion of this study is that activation of MORs has a complex influence on striatal LTSIs, which is mediated through a combination of direct inhibitory effects, a decrease of cholinergic tone and a decrease of GABAergic tone. As a result, MOR activation can cause either strong hyperpolarizations or strong depolarizations in LTSIs, in a manner that reflects the relative contribution of these three events to the excitability of individual interneurons. When GABAergic influences was pharmacologically removed, the effects of DAMGO were invariably inhibitory, showing that release from GABAergic inhibition is required for the expression of depolarizing MOR-mediated effects. On the other hand, dual effects were still present in the presence of the nicotinic receptor antagonist mecamylamine, but not in the presence of the muscarinic receptor antagonist atropine. This finding shows that release of LTSIs from muscarinic inhibition was also required for depolarizing DAMGO effects. This complex picture can be explained by considering the fact that MORs, muscarinic, nicotinic and GABA_A_ receptors are all present postsynaptically in LTSI, while MORs (Figure 7) also inhibit cholinergic terminals. The net effects on a certain LTSI will therefore depend on the relative balance of direct and indirect (cholinergic and GABAergic) influences, that can result in either net excitation or net inhibition. The ability of ACh to modulate GABA release (Elghaba et al., 2016) also needs to be considered. The influence of GABA and ACh (apparently released in a spike-independent manner) persisted in the presence of TTX, although the balance of excitatory and inhibitory factors was altered, as depolarizations were significantly more frequent than in control solution. A surprising finding of the present study was that, in contrast to previous observations on striatal projection neurons (Miura et al., 2007, 2008), GABA release on LTSIs was not directly affected by MORs, although indirect effects caused by MOR through reduction in ACh release were clearly present. In such previous studies, the effects of MORs on GABA release were studied in the absence of ACh receptor antagonists. Thus, these differences could either reflect a genuine specificity of responses to MOR activation in LTSIs vs. projection neurons or, alternatively, it is possible that a similar indirect modulation of GABA inputs by MOR was present in striatal projection neurons but not detected in previous studies. Further experiments will be required to clarify this important issue.

The most likely endogenous activator of striatal MORS is enkephalin, that is released in the striatum by projection neurons of the so-called indirect pathway (Gerfen and Surmeier, 2011). The present findings complement previous observations on how LTSIs inhibit projection neurons (Tepper et al., 2008; Straub et al., 2016), are involved in mutual excitation with cholinergic interneurons (Elghaba et al., 2016) and are modulated in opposite directions by dopamine and serotonin (Centonze et al., 2002; Cains et al., 2012). The emerging picture for the striatal microcircuit is one where these interneurons are controlled by extrinsic neuromodulators and are involved in complex interactions with projection neurons and other interneurons. It is evident that in order to unravel how this complex communication shapes striatal neuronal dynamics, computational models of striatal networks will have to be upgraded to incorporate the newly discovered interactions.

The striatum comprises two compartments, named striosome and matrix, that differ significantly in the density of MOR expression (Graybiel and Ragsdale, 1978; Friedman et al., 2015). As in the present experiments we did not identify the striatal compartments in which the LTSIs were located, we cannot exclude that the location of the LTSIs in the striosome-matrix mosaic could be a factor in their responses to DAMGO. However, previous studies have suggested that the major differences in MORs distribution in the two compartments are in terms of their presence on glutamatergic terminals rather than GABAergic neurons (Miura et al., 2007). As the present experiments were carried out in the presence of glutamate receptor blockers, it seems unlikely that the differences observed can be attributed to location of LTSIs in the matrix or striosome compartments.

The present experiments were carried out in brain slices using an exogenous MOR agonist. In the intact striatum, LTSI MORs are likely to be activated by enkephalin released by striatopallidal projection neurons. The GABAergic inputs to LTSI would be presumably much larger* in vivo* than *in vitro*, as a consequence of dynamic excitation of GABAergic neurons. While cholinergic interneurons are spontaneously active in brain slices (Bennett et al., 2000), most GABAergic neurons are not (Tepper et al., 2010). Thus, it is likely that the effects of enkephalin on GABA inputs is proportionally more dramatic *in vivo* than *in vitro* when compared to the effects on cholinergic inputs. This different balance is likely to favor the excitatory effects of enkephalin on LTSIs in behaving animals.

In addition to their ability to influence other striatal neurons through GABA, somatostatin and nitric oxide (Kawaguchi, 1993; Tepper et al., 2010; Elghaba et al., 2016), LTSIs are likely to play an important role in neurovascular coupling through release of nitric oxide (Duchemin et al., 2012). These interneurons are indeed the only neuronal type known to express nitric oxide synthase (Kawaguchi, 1993). The potential ability of MOR agonists to affect neurovascular coupling is fascinating both in physiological terms and in the context of opioid abuse, given that drugs such as heroin and morphine act primarily as MOR agonists. The present results provide a cellular basis for further investigations of these issues in the wider context of how neuronal activity is linked to blood circulation in the brain in physiological and pathological conditions.

## Author Contributions

RE carried out the experiments, analyzed the experimental data, co-designed the experimental protocols and co-wrote the manuscript. EB co-designed the experimental protocol, co-wrote the manuscript and took part in data analysis.

## Conflict of Interest Statement

The authors declare that the research was conducted in the absence of any commercial or financial relationships that could be construed as a potential conflict of interest.
